# Unraveling the Role of CYP2E1 in Antitubercular Drug–Induced Hepatotoxicity: From Molecular Mechanisms to Clinical Implications

**DOI:** 10.1155/ijh/9980298

**Published:** 2025-11-20

**Authors:** Debasree Bishnu, Suman Santra, Swagata Purkait, Amal Santra

**Affiliations:** ^1^Department of Basic Science and Disease Biology, John C. Martin Center for Liver Research & Innovations, Kolkata, West Bengal, India; ^2^Department of Hepatology, Center for Liver Research, Institute of Post Graduate Medical Education & Research, Kolkata, West Bengal, India

**Keywords:** antitubercular drugs, cytochrome P450 2E1, drug induced liver injury, genetic polymorphism, hepatocellular damage, oxidative stress

## Abstract

Antitubercular (AT) drugs, particularly isoniazid (INH), rifampicin (RIF), pyrazinamide (PYZ), and ethambutol (EMB), are the cornerstone of tuberculosis (TB) treatment. However, their use is often limited by the risk of hepatotoxicity, a potentially severe side effect. Among the factors implicated in drug-induced liver injury, cytochrome P450 2E1 (CYP2E1) is emerging as a key enzyme in the pathogenesis of hepatotoxicity. CYP2E1 is involved in the oxidative metabolism of many xenobiotics, including AT drugs, and is known to produce reactive oxygen species (ROS) during the metabolism process, which can lead to cellular damage. This review investigates the potential role of CYP2E1 in the mechanisms behind AT drug–induced hepatotoxicity and explores the biochemical and molecular pathways through which CYP2E1 might contribute to liver injury. Genetic polymorphisms in the CYP2E1 gene, which affect its activity, may also play a role in individual susceptibility to AT drug–induced hepatotoxicity. This review also deals with how multifactorial interactions including genetic polymorphisms in CYP2E1, N-acetyl transferase 2 (NAT2), and glutathione-S-transferase (GST), as well as factors such as drug–drug interactions, nutritional status, coexisting infections (e.g., hepatitis B/C), and alcohol consumption collectively modulate individual susceptibility to AT drug–induced hepatotoxicity. By elucidating the role of CYP2E1 in AT drug–induced hepatotoxicity, this review provides a foundation for future therapeutic strategies, including the development of safer drug formulations or adjunct therapies targeting CYP2E1 to mitigate hepatotoxicity.

**Highlight**

• Cytochrome P450 2E1 (CYP2E1) plays a central and multifaceted role in AT drug–induced hepatotoxicity.
• CYP2E1 contributes to liver injury by bioactivating INH into reactive intermediates.
• Coadministration with RIF amplifies CYP2E1 activity and increases hepatotoxic potential.
• INH itself and its metabolites can form adducts with CYP2E1 that may provoke immune-mediated liver injury.
• Genetic polymorphisms in CYP2E1, especially the C1/C1 genotype, significantly heighten individual susceptibility to AT drug–induced liver damage.

## 1. Introduction

Drug-induced liver injury (DILI) is now recognized as a cause of both acute and chronic liver diseases. The type of drugs causing liver injury has changed over time. Epidemiological studies showed that in the 20th century, chlorpromazine, isoniazid (INH), amoxicillin, and cimetidine were the Top 4 drugs causing DILI [1]. Currently, in Western countries, amoxicillin/clavulanic acid represents the leading cause of DILI [2], whereas in Eastern regions, combinations of antitubercular (AT) drugs are the major contributors [3]. There is marked geographic variability in the class of drug responsible for causing idiosyncratic DILI. Acetaminophen toxicity causes acute liver failure (ALF) in the West [4], but it is only around 1% of all causes of DILI-related ALF in India [5]. Several large and well-characterized DILI registries, based on prolonged follow-up of well-characterized acute DILI subjects, have shown that chronic hepatitis can occur as a distinct outcome in DILI [6–8]. Thus, DILI is increasingly being recognized not only as a cause of clinically significant acute liver disease but also as chronic liver disease. Some recent studies have explored the role of AT drugs as not only the leading cause of ALF but also the second most common cause of drug-induced acute on chronic liver failure in India [9, 10]. Prolonged medication and inadvertent rechallenge might be risk factors for developing chronic DILI [11].

The clinical characteristics and outcomes of DILI are well documented; however, the molecular mechanisms underlying DILI, particularly antitubercular drug–induced liver injury (AT-DILI), remain incompletely understood. Among the molecular mechanisms implicated in AT-DILI, the cytochrome P450 (CYP) enzyme system, particularly the CYP2E1 isoform, has garnered increasing attention. CYP2E1 is known for its role in the oxidative metabolism of a wide range of drugs, including AT medication [12]. This enzymatic activity leads to the generation of reactive oxygen species (ROS), which can initiate oxidative stress, lipid peroxidation, and mitochondrial dysfunction, all hallmarks of oxidative damage induced hepatocellular injury [13, 14]. Moreover, genetic polymorphisms in the CYP2E1 gene may contribute to interindividual variability in susceptibility to hepatotoxicity, making it a key factor in personalized risk assessment [15].

This review is aimed at exploring the mechanistic role of CYP2E1 in AT drug–induced hepatotoxicity, highlighting how it interacts with other metabolic pathways and host factors. By dissecting the biochemical, genetic, clinical, and experimental evidence, we aim to provide a comprehensive framework for understanding CYP2E1's role in AT-DILI and identifying potential strategies for risk mitigation and therapeutic intervention.

### 1.1. Prevalence of DILI

Estimating the true prevalence of DILI remains a challenge due to inconsistencies in diagnostic criteria, reporting systems, and healthcare infrastructure across regions. Despite this, several population-based studies have attempted to quantify its occurrence; the prevalence of DILI is widely varied in different parts of the world.  Table 1 summarizes the available data in the literature.

DILI is a rare adverse drug reaction that accounts for less than 1% of acute liver injury [22, 27]. Despite its rarity, DILI is the leading cause of ALF with a case fatality rate ranging from 10% to 50% [27, 28]. In contrast, acetaminophen, a commonly used analgesic and antipyretic is the most common cause of ALF (46%), followed by cases of indeterminate DILI (15%) and idiosyncratic DILI (12%) [27].

#### 1.1.1. Prevalence of AT-DILI

Although tuberculosis (TB) is a global problem, Southeast Asia and Africa have a high TB burden accounting for 68% of global infections. India shares the largest TB cases at around 28% of the global infection rate [29, 30]. Most of the first-line drugs such as INH, rifampicin (RIF), and pyrazinamide (PYZ) used for TB eradication are potential hepatotoxic agents [3, 18, 31] and are commonly implicated in both adult and pediatric DILI cases due to their hepatotoxic potential [32]. AT drugs, particularly INH, are well known for causing liver injury in about 0.1%–1% of users [33]. The hepatotoxicity potential of these drugs is escalated when they are used in combination. A review of US FDA data estimated that 23.2 per 100,000 individuals die from hepatotoxicity while receiving INH prophylactic therapy [34]. A meta-analysis further showed that INH alone increases the risk of liver injury **(**OR 1.6**)**, and this risk is significantly higher when combined with RIF **(**OR 2.6**)**, underscoring the elevated hepatotoxic potential of combination therapy [33, 35, 36].

AT-DILI varies significantly across regions, influenced by differences in TB prevalence, treatment practices, genetic susceptibility, and healthcare infrastructure. AT-DILI remains a major global health concern, particularly in regions with a high burden of TB. The global incidence of AT-DILI varies considerably across studies, with reported rates ranging from approximately 2% to 28% [35, 37, 38], reflecting differences in diagnostic definitions, patient demographics, and treatment regimens. A recent systematic review and meta-analysis highlighted the geographical variability, with rates as low as 1.13% in Italy and as high as 35.07% in Uganda [39]. These disparities emphasize the need for region specific surveillance strategies and a deeper understanding of the mechanisms driving AT-DILI.

Western countries such as the United Kingdom, France, and Iceland report much lower rates of AT-DILI [25, 40]. For example, AT drugs account for only 5.9% of DILI cases in the United States, 5.8% in Latin America, and 7.6% in Spain [41]. The lower incidence in these regions is partly due to the less frequent use of multidrug AT regimens, as INH is often used alone for latent TB prophylaxis.

In India and China, AT medications are major contributors to DILI, with INH, RIF, and PYZ commonly involved. In India, around 12.6% of TB patients develop DILI when treated with four first-line AT drugs. AT drugs are major contributors to DILI, accounting for approximately 46%–58% of cases in India [42] and 21.9% of cases in China [17]. In India, DILI from these drugs is also a leading cause of ALF, reflecting the substantial hepatic burden imposed by TB treatment. Alarmingly, these combination regimens are responsible for 63%–72% of drug-induced ALF cases in the country [18, 42].

In a large cohort of 460 pediatric DILI cases, AT drugs were identified as one of the leading contributors, accounting for 13.9% of cases, second only to methotrexate (13.5%), an antineoplastic agent. This underscores the significant role of AT therapy in pediatric hepatotoxicity, especially when used as part of multidrug regimens in TB endemic regions [43].

### 1.2. Comprehensive Overview of Drug Properties and Metabolism

**Figure 1:**
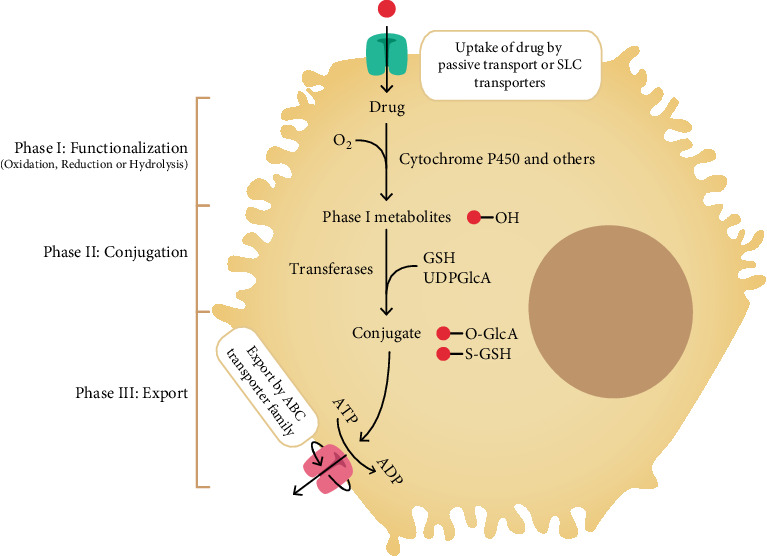
Schematic representation of drug metabolism pathways encompassing Phase I, Phase II, and Phase III processes. Following administration, the parent drug undergoes Phase I reactions (functionalization), which include oxidation, reduction, or hydrolysis. The oxidation reaction is primarily mediated by CYP enzymes. These reactions introduce or expose functional groups on the drug molecule, often producing reactive or moderately polar metabolites. Phase II reactions (conjugation) follow, in which these metabolites are conjugated with endogenous hydrophilic moieties such as UDP-glucuronic acid, glutathione (GSH) through the action of transferase enzymes, resulting in more water-soluble and typically inactive metabolites. Phase III involves the active transport and excretion of these conjugated metabolites via efflux transporters (ABC transporter family) into bile, urine, or feces, completing the elimination process. SLC transporter, Solute carrier transporter; GSH, glutathione; UDPGlcA, UDP-glucuronic acid; ABC, ATP-binding cassette.

The liver is the primary site for drug metabolism and biotransformation due to its high concentration of enzymatic systems and its strategic anatomical position between the gastrointestinal tract and systemic circulation [44]. The efficiency of hepatic drug metabolism can be compromised by various factors: threshold dose; physicochemical properties like lipophilicity, uptake by hepatocytes, and metabolism; and the formation of reactive metabolites during biotransformation. These metabolites may induce oxidative stress, mitochondrial dysfunction, and inhibition of hepatic transporters, thereby contributing to DILI [45]. Chen et al. reported a high dose of drug (100 mg or more) and high lipophilicity of logP more than 3 was found to be related to DILI [46]. Most drugs are taken up into hepatocytes either passively or through solute carrier transporters expressed on the hepatocyte membrane [47, 48]. Once inside the cell, the metabolic processing of drugs occurs in three main phases as depicted in Figure 1 [44].

Phase I reactions typically involve oxidation, reduction, or hydrolysis. The CYP family of enzymes plays a pivotal role in this phase as the oxidase enzyme. The resulting metabolites of this phase are often more reactive and may possess altered biological activity compared to the parent compound [44, 49]. While these transformations increase solubility, they may also produce chemically unstable intermediates. Some of these reactive metabolites can bind covalently to cellular proteins, forming drug–protein adducts [50–52].

Phase II metabolism involves the conjugation of the parent compound or its Phase 1 metabolites with endogenous molecules such as glucuronic acid, sulfate, or GSH [49, 51]. These conjugation reactions generally serve to detoxify the compound and increase its aqueous solubility, thereby facilitating excretion.

In Phase III, transport proteins located on the canalicular and sinusoidal membranes mediate the efflux of conjugated metabolites into bile or blood [51, 53]. These include members of the ATP binding cassette transporter family. Inhibition of these transporters can impair biliary excretion, leading to the retention of bile salts and other compounds that may damage hepatocyte organelles, particularly mitochondria [54, 55].

### 1.3. Classification of DILI

Based on different features as depicted in Table 2, DILI can be classified into intrinsic and idiosyncratic types. In intrinsic forms of hepatotoxicity, the direct toxic effects of the parent compound or its metabolites predominate. Mitochondrial dysfunction, oxidative stress, endoplasmic reticulum (ER) stress, and inhibition of bile acid transporters are frequently observed [56, 57]. Accumulation of toxic intermediates can result in loss of mitochondrial membrane potential, depletion of ATP, and activation of cell death pathways, including necrosis and apoptosis [58].

In contrast, idiosyncratic DILI is thought to involve complex interactions between drug-derived antigens and host immune responses. Genetic predisposition, particularly in human leukocyte antigen variants, may influence antigen presentation and T cell activation [59]. However, the majority of individuals with risk alleles do not develop liver injury, suggesting that additional environmental or metabolic triggers are required [60].

Importantly, these mechanistic categories are not mutually exclusive. Many hepatotoxic drugs exhibit overlapping features of direct toxicity and immune-mediated injury. The relative contribution of each pathway may vary depending on the chemical structure of the compound, the dose, and individual susceptibility factors. Understanding these diverse mechanisms is essential for improving the prediction, diagnosis, and prevention of DILI in clinical practice.

### 1.4. Manifestation of DILI

DILI can present in a range of clinical and biochemical forms, making its recognition difficult in the absence of high clinical suspicion. The manifestations vary from isolated asymptomatic elevations in liver enzymes to fulminant liver failure. In many cases, symptoms are nonspecific such as fatigue, malaise, nausea, and abdominal discomfort, which may precede any overt biochemical changes. Jaundice, pruritus, dark urine, and pale stools suggest more advanced involvement [61].

Biochemically, DILI is classified based on the pattern of liver injury into hepatocellular, cholestatic, or mixed types. This classification is guided by the ratio of serum alanine aminotransferase to alkaline phosphatase, expressed as fold elevations above the upper limit of normal [62]. Hepatocellular injury is associated with higher transaminase levels and carries a greater risk of ALF [63, 64]. Cholestatic patterns tend to have a more protracted course but often resolve completely. The mixed pattern combines features of both and may complicate clinical interpretation [62].

In some cases, DILI resembles other liver diseases. A subset of patients shows features mimicking autoimmune hepatitis, including the presence of autoantibodies and hypergammaglobulinemia [65]. Others may develop hypersensitivity reactions, presenting with fever, rash, eosinophilia, or lymphadenopathy [66, 67]. Rarely, patients develop vanishing bile duct syndrome, nodular regenerative hyperplasia, or drug-induced fatty liver disease [68].

The severity of DILI spans a broad spectrum. While many patients recover completely after discontinuation of the offending agent, a small proportion progresses to ALF, requiring transplantation [64, 65]. Chronic liver injury is also possible, although less common [68].

## 2. Exploring CYP: The Enzymatic Super Family Behind Drug Reactions

CYP enzymes play a pivotal role in the Phase I drug metabolism reaction, functioning as terminal oxidases. These heme-containing enzymes are not only involved in the metabolism of exogenous drugs but also act on a wide range of endogenous substrates, such as steroids, fatty acids, and bile acids [69]. Although predominantly localized in the liver, this superfamily of enzymes is also significantly expressed in several other tissues and organs [70]. The catalytic cycle of CYP enzymes relies on NADPH-CYP reductase. In this process, CYP enzymes use the electron from NADPH to form a ferryl-oxo intermediate, a highly reactive species responsible for substrate oxidation. While CYP-mediated metabolism typically results in detoxification, certain substrates such as carbon tetrachloride and acetaminophen can undergo bioactivation, leading to the generation of ROS and oxidative tissue damage [71–73].

Among the different human CYP isoforms, six (CYP1A2, CYP2C9, CYP2C19, CYP2D6, CYP2E1, and CYP3A4) account for nearly 90% of drug metabolism [74]. While CYP2E1 metabolizes only a small fraction of clinically prescribed drugs, it plays a disproportionately large role in DILI due to its high capacity for generating ROS and bioactivating toxic intermediates.

### 2.1. Mechanistic Insights Into CYP2E1 Induced Oxidative Damage

CYP2E1 is a ~57 kDa enzyme predominantly expressed in hepatocytes, where it is primarily anchored to the membrane of the smooth ER. Smaller amounts are also localized in mitochondria, plasma membrane, and in liver nonparenchymal cells such as Kupffer cells [72]. Although CYP2E1 metabolizes only about 2% of clinically prescribed drugs, its key substrates include acetaminophen, the AT drug INH, ethanol, and chlorzoxazone [75].

CYP2E1 is often referred to as a “leaky enzyme” because its heme iron remains in a high-spin state even in the absence of a substrate, allowing continuous electron transfer and contributing to unregulated ROS production. Although this dormant ROS production can be compensated by intracellular GSH levels [76], recent studies suggest that in environments enriched with hydrogen peroxide and nonheme iron, CYP2E1 facilitates the formation of reactive hydroxyl radicals which in turn facilitates ethanol-induced oxidative stress [77, 78]. Besides, it is believed to play a key role in INH-induced hepatotoxicity. Its expression is inducible by various substrates, including INH, pyrazole, and ethanol [79]. Notably, CYP2E1 activity can be significantly upregulated when INH is coadministered with RIF, potentially increasing the risk of liver injury.

### 2.2. Mitochondrial Cytochrome P450 2E1 (mtCYP2E1): An Emerging Player in Hepatotoxicity

Although microsomal CYP2E1 is well established as a key enzyme in drug biotransformation, growing evidence of mitochondrial dysfunction in DILI has shifted attention toward mtCYP2E1. While CYP2E1 has been extensively studied in the ER, its mitochondrial counterpart may contribute uniquely to oxidative stress and liver damage due to its location within the organelle most vulnerable to redox imbalance [80]. In rat models, this mitochondrial isoform appears as a truncated variant of the microsomal form, approximately 40 kDa in size, lacking part of the amino-terminal [81, 82]. However, other studies have reported a larger, phosphorylated 52 kDa form of CYP2E1, with mitochondrial translocation mediated by protein kinase A–dependent phosphorylation [70]. Unlike its microsomal counterpart, the catalytic activity of mtCYP2E1 relies on mitochondrial adrenodoxin reductase and adrenodoxin, rather than NADPH-CYP reductase [83].

Studies have shown that both truncated and full-length versions of mtCYP2E1, when expressed in HepG2 or COS-7 cell lines, lead to increased ROS, reduced mitochondrial membrane potential, and greater oxidative damage compared to endoplasmic reticulum–localized cytochrome P450 2E1 (erCYP2E1), particularly under stress conditions like GSH depletion or ethanol exposure [76, 84]. While both mtCYP2E1 and erCYP2E1 can elevate ROS levels, mitochondrial localization appears to result in more severe cellular toxicity. In vivo studies, including diabetic and ethanol-treated animal models, further support a role for mtCYP2E1 in oxidative liver injury, though the specific contributions of mitochondrial versus microsomal CYP2E1 remain difficult to separate [85, 86]. Importantly, the level of mtCYP2E1 varies significantly among individuals, potentially due to genetic polymorphisms in the targeting sequence, and this variability may influence susceptibility to drug-induced liver damage [87]. Collectively, these findings suggest that mtCYP2E1 plays a distinct and potentially more harmful role in hepatotoxicity compared to its microsomal counterpart. But currently there is no direct evidence linking mtCYP2E1 to AT drugs–induced cytotoxicity.

### 2.3. Role of CYP2E1 in Liver Diseases

CYP2E1 is critically important in the overall landscape of hepatotoxicity as it plays a central role in the pathogenesis of several liver diseases [78, 88]. While it constitutes only a small fraction of total hepatic CYP enzymes, CYP2E1's high inducibility, especially by ethanol, high-fat diets, and certain drugs, allows it to exert disproportionate toxic effects. Additionally, individual variability in CYP2E1 expression, genetic polymorphisms, and its subcellular distribution, especially its mitochondrial form (mtCYP2E1), can further influence susceptibility to liver injury. Stephens et al. classified CYP2E1 into three genotypes—two homozygous genotypes (C1/C1 and C2/C2) and one heterozygous genotype (C1/C2) based on variations at the Rsa I restriction site [89]. Among these, individuals with the CYP2E1 C1/C1 genotype have been found to exhibit an increased risk of hepatotoxicity, particularly in cases of DILI and hepatocellular carcinoma (HCC) [90, 91]. Overall, CYP2E1 is a central mediator of hepatotoxicity and represents a critical target for both therapeutic strategies and risk assessment in liver injury.

CYP2E1 plays a significant role in DILI by metabolizing various drugs into reactive and toxic intermediates. In both intrinsic and idiosyncratic DILIs, CYP2E1 is responsible for converting otherwise inert or mildly toxic substances into highly reactive intermediates, such as the formation of NAPQI during acetaminophen metabolism or hydrazine during INH metabolism, which can lead to cellular stress, GSH depletion, and hepatocyte death [12]. Besides, CYP2E1 contributes to liver damage induced by chemicals like thioacetamide, vinyl chloride, and carbon tetrachloride [12, 92, 93].

CYP2E1 also plays a crucial role in the development and progression of alcoholic liver disease due to its high inducibility and catalytic efficiency for ethanol [12, 94]. This enzyme generates large amounts of ROS and toxic metabolites such as acetaldehyde, leading to oxidative stress, lipid peroxidation, mitochondrial dysfunction, and inflammation which collectively damage liver cells and promote fat accumulation (steatosis) leading to steatohepatitis, fibrosis, cirrhosis, and even HCC [95]. Chronic alcohol intake enhances CYP2E1 activity by stabilizing the enzyme and preventing its degradation via the proteasome. This increase in CYP2E1 has been shown to aggravate oxidative liver damage, whereas its inhibition or genetic knockout offers protection against alcohol-induced liver injury. [72, 96].

CYP2E1 has been implicated in the progression of nonalcoholic fatty liver disease (NAFLD) to its more severe form, nonalcoholic steatohepatitis (NASH), which involves inflammation and fibrosis [97]. High-fat diets, as well as fasting or prolonged starvation, can increase CYP2E1 levels. Although the mechanisms remain unclear, CYP2E1-generated ROS and toxic lipid intermediates are suspected contributors. In obese patients, a correlation has been observed between CYP2E1 expression and lipid peroxidation, indicating CYP2E1's role in promoting oxidative stress. Despite elevated serum GSH, patients with steatosis or steatohepatitis exhibit reduced hepatic GSH, likely due to fatty acid–induced toxicity [98].

### 2.4. Role of CYP2E1 in AT Drug Metabolism and DILI

AT drugs, such as INH, RIF, and PYZ are essential components of first-line TB treatment [34, 35, 99, 100]. However, their clinical use is often complicated by DILI, which can range from mild elevations in liver enzymes to severe, life-threatening hepatotoxicity. Among these agents, INH is most strongly associated with hepatotoxic effects, particularly when metabolized into toxic intermediates [38, 101–103]. A key player in this process is CYP2E1, an enzyme known for its role in oxidative metabolism and the generation of ROS [104–106].

#### 2.4.1. INH

**Figure 2:**
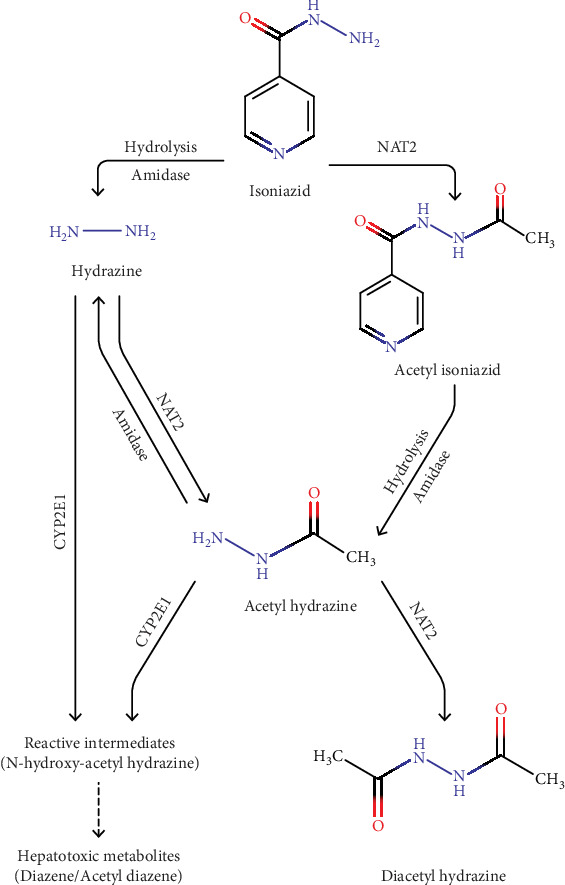
Overview of isoniazid (INH) Metabolism. This figure illustrates the metabolic pathways of INH in the liver. INH primarily undergoes acetylation by N-acetyltransferase 2 (NAT2) to form acetyl INH, which can be further converted to acetyl hydrazine. Alternatively, INH may undergo hydrolysis by amidase to yield hydrazine. Both hydrazine and acetyl hydrazine serve as substrates for CYP2E1, leading to their oxidative conversion into N-hydroxy metabolites. These intermediates undergo subsequent dehydration to generate diazene, a highly reactive and hepatotoxic species.

In the liver, INH is mainly metabolized by two key enzymes, N-acetyltransferase 2 (NAT2), and CYP2E1. NAT2 converts INH into acetyl INH, which is further hydrolyzed into acetyl hydrazine by amidase. This compound can be detoxified into a harmless form diacetyl hydrazine by another acetylation reaction. INH can also be directly hydrolyzed into a liver-damaging substance, hydrazine by amidase. Acetyl hydrazine and hydrazine, both can undergo CYP2E1-mediated oxidation to form N-hydroxy-acetyl hydrazine, which then dehydrates to acetyl diazene, a potent hepatotoxic metabolite (Figure 2). In people who are slow acetylators where NAT2 works less efficiently, this toxic pathway becomes much more active, especially when INH is taken together with RIF, greatly increasing the risk of liver injury [34, 107, 108].

INH, administered at 300 mg daily, is a well-known cause of DILI involving multiple damaging pathways. It generates reactive metabolites that can directly damage hepatocytes and contribute to immunoallergic injury in genetically predisposed individuals, particularly those with the HLA-DQB1∗0201 allele. At the same time, INH disrupts mitochondrial function damaging the cell's energy factories which results in energy loss and cell death. The body's usual protective antioxidant systems, like the Nrf2 pathway, fail to respond properly, making the damage worse [109]. Its hydrazine derivatives may also inhibit histone deacetylase, potentially disrupting gene regulation and impairing liver cell regeneration. These combined effects highlight the multifactorial and potentially severe hepatotoxic potential of INH [110–112].

There is growing evidence that the immune system plays a key role in INH-induced liver injury. Some patients develop autoimmune conditions like lupus during therapy, and elevated Th17 cells, along with lymphocyte responses to INH-modified proteins, have been observed. In severe cases, antibodies against INH and CYP2E1, particularly IgG3, suggest both cellular and antibody-mediated immune responses contribute to liver inflammation and damage [113–117].

Genetic polymorphism of CYP2E1 also plays a very critical role in INH-mediated hepatotoxicity. Individuals with the CYP2E1 C1/C1 genotype show increased enzyme activity, leading to enhanced production of toxic metabolites from INH. The risk of hepatotoxicity becomes exacerbated in the presence of NAT2 slow acetylator variants [90].

#### 2.4.2. RIF

RIF itself is less likely to cause liver injury when used alone, but it becomes more toxic when combined with other AT drugs, especially INH. This is because RIF is a strong inducer of liver enzymes, particularly CYP2 family [118], by activating a protein called the pregnane X receptor in liver cells [119]. This speeds up the metabolism of INH, increasing the production of its toxic byproducts. RIF induces INH hydrolases, leading to elevated production of hydrazine, especially in individuals who are slow acetylators [34, 120]. In addition to these effects, RIF can cause temporary unconjugated hyperbilirubinemia by interfering with how the liver takes up bilirubin. In some cases, it may also cause conjugated hyperbilirubinemia by blocking the bile salt export pump, which helps move bile out of liver cells [121–123]. RIF also interacts with drug transport proteins like ABCB1, and genetic variants such as ABCB1 3435T have been linked to higher DILI risk, particularly in patients receiving both AT therapy and antiretroviral therapy [124, 125].

#### 2.4.3. PYZ

PYZ is considered the most hepatotoxic among the first-line AT drugs. Hepatocellular damage by PYZ is mediated by amidase and xanthine oxidase to its two toxic metabolites: pyrazinoic acid and 5-hydroxy pyrazinoic acid [126]. Though there is no clear evidence of CYP2E1 behind PYZ-induced hepatotoxicity, it is found to be associated with both intrinsic as well as idiosyncratic DILI and has a relatively long half-life in people with preexisting chronic liver disease [126, 127].

### 2.5. The Mechanism Involved in CYP2E1 Mediated AT-DILI

AT-DILI injury involves both direct toxic effects and idiosyncratic mechanisms. Toxic metabolites of AT drugs can covalently bind to biological macromolecules, leading to cellular dysfunction and liver damage [128]. There is strong evidence of oxidative stress, characterized by increased lipid peroxidation.

During the preclinical phase of drug development, male Wistar rats treated with INH at 100 mg/kg have demonstrated clear evidence of liver injury, mirroring the hepatotoxic potential seen in humans. A key factor in this process is the bioactivation of INH by CYP2E1, which metabolizes INH into reactive intermediates, including hydrazine, a known hepatotoxin [129].

Another experimental evidence from in vivo models has reinforced the central role of CYP2E1-mediated oxidative stress in AT-DILI. In a murine study, cotreatment with INH and RIF significantly increases hepatic oxidative stress, largely mediated by the enzyme CYP2E1. While individual treatment with INH or RIF leads to depletion of hepatic GSH, their combination exacerbates GSH loss and increases oxidized glutathione (GSSG), indicating elevated oxidative stress. This stress extends to the mitochondria, as shown by reduced mitochondrial GSH and increased mitochondrial GSSG. Importantly, CYP2E1 expression and activity were markedly induced by INH-RIF cotreatment showing a 2.2-fold increase supporting its central role in generating ROS and promoting lipid peroxidation and protein oxidation. These findings highlight CYP2E1 as a key mediator in INH-RIF-induced hepatotoxicity [14].

A pivotal role for CYP2E1 in the development of chronic DILI and fibrosis was demonstrated in a long-term in vivo study examining the effects of combined INH (50 mg/kg) and RIF (100 mg/kg) administration. In this study, BALB/c mice exposed to INH-RIF over 4–24 weeks exhibited a marked decrease in hepatic GSH levels and an increase in lipid peroxidation, supporting the presence of sustained oxidative stress during INH-RIF treatment. Progressive hepatic fibrosis developed, characterized by hepatocyte apoptosis, activation of hepatic stellate cells (HSCs), and increased collagen deposition. Notably, these fibrotic changes were accompanied by a marked increase in hepatic CYP2E1 expression and NADPH oxidase (NOX) activity, both of which contributed to sustained oxidative stress within the liver. These findings suggest that CYP2E1 acts upstream in the fibrogenic cascade by generating ROS in response to INH metabolism, which in turn promotes HSC activation via NOX-driven pathways. Together, these findings underscore CYP2E1's pivotal role in initiating and perpetuating oxidative stress during INH-RIF exposure, thereby promoting hepatocellular injury and fibrogenic signaling [13].

The role of CYP2E1 in HSC activation and fibrogenesis in response to INH exposure was further investigated using the human HSC line LX2. The study found that INH alone could increase CYP2E1 levels and NOX activity over 72 h, leading to increased oxidative stress and depletion of intracellular GSH. While these changes were sufficient to induce *α*-smooth muscle actin (*α*-SMA), a marker of HSC activation, they did not directly upregulate collagen1A1 (COL1A1) expression. However, when LX2 cells were pretreated with pyrazole to induce CYP2E1 overexpression, INH exposure resulted in significantly elevated NOX activity, ROS, *α*-SMA, and collagen type I expressions. These findings indicate that CYP2E1, although present at low basal levels in HSCs, plays a pivotal role in mediating INH-induced fibrogenic responses when overexpressed. The data underscore that CYP2E1-facilitated oxidative stress can act as a secondary mechanism by which INH indirectly contributes to hepatic fibrosis via activation of stellate cells and stimulation of collagen production [130].

In an in vitro investigation aimed at understanding CYP2E1-mediated AT drug–induced hepatotoxicity, researchers utilized E47 cells, a HepG2-derived cell line that overexpresses CYP2E1, and C34 cells, the corresponding control line with negligible CYP2E1 expression. The study demonstrated that pharmacological upregulation of heme oxygenase-1 (HO-1), an antioxidant enzyme significantly reduced cell death in both cell lines following exposure to INH and RIF. Notably, the protective effect of HO-1 was more pronounced in E47 cells, highlighting the role of CYP2E1-mediated oxidative stress in enhancing hepatocyte susceptibility to drug-induced injury. These findings suggest that upregulation of HO-1 could serve as a potential therapeutic strategy to counteract CYP2E1-associated liver toxicity during AT therapy [131].

Recent evidence challenges the previously held notion that INH induced hepatotoxicity is entirely nonimmune-mediated. The study revealed that a significant number of patients with INH induced liver failure developed antibodies not only against INH itself but also against CYP2E1. INH was shown to form covalent adducts with CYP2E1, which likely alters the enzyme's structure, making it immunogenic and triggering the production of anti-CYP2E1 antibodies. These autoantibodies were absent in INH treated individuals without liver injury, indicating that CYP2E1 modification by INH may play a critical role in the pathogenesis of immune-mediated liver damage. This finding positions CYP2E1 as a key player not only in the metabolic activation of INH but also in the initiation of immune responses contributing to idiosyncratic DILI [117].

Another study reinforces the critical involvement of CYP2E1 in INH-induced hepatotoxicity, particularly when combined with inflammatory stimuli such as lipopolysaccharide (LPS). This model mimics a clinically relevant scenario where underlying infections or immune activation may exacerbate drug toxicity. The study demonstrated that both INH and LPS independently induce CYP2E1 expression and that their combination synergistically amplifies oxidative stress, lipid peroxidation, and bile acid dysregulation. Notably, while administration of diallyl sulfide (DAS), a CYP2E1 inhibitor partially mitigated liver damage, full protection was only achieved when DAS was co-administered with dexamethasone (DEX), an anti-inflammatory agent. This finding suggests that CYP2E1 activity alone does not fully account for hepatotoxicity, but rather interacts with inflammatory pathways to enhance liver injury. These results underscore the dual role of CYP2E1-mediated ROS generation and immune-mediated liver sensitivity, and support the hypothesis that CYP2E1 is a key contributor to the hepatotoxic potential of AT drugs, especially in the presence of coexisting inflammation [106].

Genetic polymorphism in CYP2E1 isoform is shown to exhibit a significant association with increased risk of AT-DILI. In a prospective study involving 318 TB patients undergoing anti-TB therapy, those with the homozygous wild-type CYP2E1 C1/C1 genotype were found to have a significantly higher risk of developing DILI compared to carriers of the mutant C2 allele. This risk was further elevated when the C1/C1 genotype was combined with the slow acetylator NAT2 genotype, suggesting a gene–gene interaction in modulating susceptibility to hepatotoxicity. Phenotypic analysis confirmed that individuals with the C1/C1 genotype had higher CYP2E1 enzymatic activity, which likely contributes to increased formation of ROS and toxic metabolites. Even after adjusting for confounders such as acetylator status and age, the CYP2E1 C1/C1 genotype remained an independent risk factor. These findings underscore the pivotal role of CYP2E1 genetic polymorphism in determining individual vulnerability to AT-DILI [90].

A recent meta-analysis of 29 studies involving 7526 individuals (1548 with AT-DILI and 5978 without) examined the relationship between CYP2E1 RsaI/PstI gene polymorphisms and AT-DILI. The findings revealed that individuals particularly those of East Asian descent with the CYP2E1 C1/C1 genotype have an increased risk of developing AT-DILI. This is because the c1/c1 genotype is associated with higher CYP2E1 enzyme activity, which reduces the inhibitory effect of INH and leads to the increased production of hepatotoxic metabolites. The results align with previous meta-analyses, supporting a genetic predisposition to liver injury from AT treatment linked to CYP2E1 variants [132].

The frequency of occurrence of the CYP2E1 PstI polymorphism (rs3813867) varies markedly across global populations, reflecting both ethnic and geographical diversity. In a study with the indigenous population of Sabah, Malaysia, Goh et al. reported the frequencies of the CYP2E1 genotypes C1/C1, C1/C2, and C2/C2 as 80.9%, 18.8%, and 0.3%, respectively [133]. The same study also provided a comparative review of the population-wise distribution of these genotypes. Among Asian populations, the C1/C1 genotype appears with a relative frequency, ranging from 46.9% in Chinese to 97.6% in South Indians. Notably, Taiwanese (63.3%), Korean (64.8%), and Chinese (73.3%, in a different cohort) populations show moderate frequencies, accompanied by the highest C2/C2 frequency (15.6%) reported in the Chinese population among Asians. In contrast, non-Asian populations, including Caucasians, Hungarians, French, and Brazilians, demonstrate a consistently high prevalence of the C1/C1 genotype, all exceeding 90%, and exhibit low frequencies of both C1/C2 and C2/C2 genotypes. The C1/C2 genotype, although present across all populations, is more prominent in certain Asian groups, such as Chinese (37.5%), Taiwanese (32.7%), and Koreans (33.1%), compared to other non-Asians. The C2/C2 genotype remains rare in most populations, with frequencies generally below 5%, except for the aforementioned Chinese subgroup [133].

CYP2E1 plays a central role in AT-DILI through both metabolic and immunological pathways. Its involvement includes the bioactivation of INH into toxic metabolites, generation of oxidative stress, promotion of hepatic fibrosis, and interaction with inflammatory responses. Additionally, genetic polymorphisms in CYP2E1, particularly the C1/C1 genotype, significantly increase individual susceptibility to hepatotoxicity. The enzyme's dual role in ROS and triggering immune-mediated responses underscores its importance as both a mechanistic contributor and a potential therapeutic target in managing AT-DILI.

## 3. Clinical Implications

Genetic polymorphisms in CYP2E1, particularly the C1/C1 genotype, are associated with increased enzymatic activity and a higher risk of INH-induced hepatotoxicity, especially in slow acetylators. Furthermore, CYP2E1 may serve as a neoantigen when covalently modified by INH, triggering autoantibody production and immune-mediated DILI. Clinically, understanding the role of CYP2E1 in AT-DILI offers critical implications for risk stratification, personalized therapy, biomarker development, and therapeutic interventions. Genetic screening, monitoring of oxidative stress markers, and adjunctive antioxidant therapies may help mitigate hepatotoxicity. Targeted modulation of CYP2E1 activity presents a promising strategy to enhance the safety of TB treatment, especially in vulnerable populations. In a nutshell, CYP2E1 stands as a central mediator of oxidative and immune-related liver injury in TB therapy. Its enzymatic properties, genetic variability, and interactions with coadministered drugs underscore the need for integrated clinical approaches to prevent, detect, and manage AT-DILI effectively.

## 4. Conclusion

CYP2E1 plays a critical and multifaceted role in the metabolism of drugs and the pathogenesis of DILI, particularly in the context of AT therapy. Although it metabolizes only a small fraction of prescribed drugs, CYP2E1 is unique in its high inducibility, inefficient electron coupling, and substantial ROS generation, which contribute significantly to hepatic oxidative stress and tissue damage.

CYP2E1's metabolic activity is a double-edged sword as it facilitates the clearance of compounds like INH but also converts them into reactive intermediates such as hydrazine, which are hepatotoxic. These metabolites can trigger direct hepatocellular damage, mitochondrial dysfunction, and immune-mediated responses. Furthermore, when coadministered with RIF, the hepatotoxic potential of INH is amplified due to enhanced CYP2E1 induction and oxidative stress.

Experimental studies in animal models and human cell lines strongly support the role of CYP2E1 in promoting lipid peroxidation, GSH depletion, stellate cell activation, and hepatic fibrosis in both acute and chronic settings. The enzyme's role in fibrosis is particularly evident through its interaction with NOX and its ability to initiate profibrotic signaling pathways in HSCs. Notably, polymorphisms in the CYP2E1 gene, especially the C1/C1 genotype, are associated with increased enzymatic activity and significantly higher susceptibility to INH-induced hepatotoxicity, particularly in slow acetylators.

Emerging data also indicate that CYP2E1 may act as a neoantigen when covalently modified by INH, leading to the production of autoantibodies and suggesting an immune-mediated component in DILI pathogenesis. This is further exacerbated in inflammatory states, such as infections or LPS exposure, where CYP2E1-driven ROS production synergizes with immune activation to intensify liver injury.

In summary, CYP2E1 is not only a key metabolic enzyme but also a central mediator of oxidative and immune-related hepatotoxicity, especially in TB therapy. Its induction by drugs like INH and RIF, the generation of toxic metabolites, the contribution to immune recognition, and its genetic variability make it a critical determinant in both dose-dependent and idiosyncratic DILI. Understanding the enzymatic, genetic, and environmental factors influencing CYP2E1 activity is essential for developing predictive biomarkers, optimizing TB therapy regimens, and identifying individuals at higher risk for liver injury. Targeted modulation of CYP2E1 activity and antioxidant strategies, such as HO-1 upregulation or CYP2E1 inhibition, may offer promising avenues for mitigating DILI in clinical practice.

## Figures and Tables

**Table 1:** Prevalence of drug-induced liver injury across different regions.

**Region**	**Estimated prevalence/incidence**	**Study type**	**Common causative drugs**
South Korea [16]	12 cases per 100,000 persons per year (more like)	Prospective nationwide study in 17 hospitals; > 70% herbal medicine-related	Traditional and herbal medicines
China [17]	23.8 cases per 100,000 persons per year	Retrospective study in general population	Herbal remedies, AT drugs
India [18, 19]	Not precisely quantified	High burden of AT drug–related ALF	AT drugs
United Kingdom [20]	2.4 cases per 100,000 persons per year	GPRD-based retrospective cohort	Amoxicillin–clavulanate, antibiotics
Sweden [21]	2.3 cases per 100,000 persons per year	Outpatient hepatology clinic-based extrapolation	Antibiotics
France [22]	~14 cases per 100,000 persons per year	Population-based prospective study (> 81,000 individuals)	Antibiotics, especially amoxicillin–clavulanate
Iceland [23]	19 cases per 100,000 persons per year	Prospective population-based registry	Amoxicillin–clavulanate (1 in 2350 users), other antibiotics
United States [24]	2.7 cases per 100,000 adults per year	Gastroenterologist-based registry; Kaiser data on ALF	Herbal and dietary supplements, antibiotics
Latin America [25]	No standardized prevalence reported	Multinational DILI registry (Spanish protocol used)	Amoxicillin–clavulanate, nitrofurantoin, cyproterone acetate
Africa [26]	Limited data available	Data mostly from HIV-positive patients on tuberculosis treatment	AT drugs

**Table 2:** Features of different types of DILI.

**Feature**	**Intrinsic DILI**	**Idiosyncratic DILI**
Predictability	Predictable	Unpredictable
Dose relationship	Dose-dependent	Not clearly dose-related (but often associated with > 50–100 mg/day)
Onset latency	Short (hours to a few days)	Variable and often delayed (days to several months)
Incidence	Relatively common at toxic doses	Rare (less than 1 in 10,000 exposed individuals)
Common examples	Acetaminophen, carbon tetrachloride	Amoxicillin–clavulanate, isoniazid, diclofenac
Genetic predisposition	Not essential	Often associated with specific HLA genotypes
Histological features	Centrilobular necrosis, minimal inflammation	Mixed patterns including cholestatic or hepatocellular inflammation
Mechanism	Direct cellular toxicity	Immune-mediated and host factor-dependent
Reproducibility in models	Reproducible in animal models	Poorly reproducible in preclinical systems
Severity	Can be severe but usually dose-limited	Can be severe or fatal, independent of dose
Risk factors	Alcohol, malnutrition, fasting	Genetic polymorphisms, age, sex, comorbidities
Drug development implications	Screened out during preclinical testing	Major cause of postmarketing drug withdrawal

## Data Availability

Data sharing is not applicable to this article as no new data were created or analyzed in this study.

## Nomenclature

TB: tuberculosis
AT: antitubercular
INH: isoniazid
RIF: rifampicin
PYZ: pyrazinamide
DILI: drug-induced liver injury
CYP: cytochrome P450
CYP2E1: cytochrome P450 2E1
mtCYP2E1: mitochondrial CYP2E1
NAT2: N-acetyltransferase 2
ROS: reactive oxygen species
ALF: acute liver failure
ER: endoplasmic reticulum
HO-1: heme oxygenase-1
GSH: glutathione
*α*-SMA: *α*-smooth muscle actin
HSCs: hepatic stellate cells
NAFLD: nonalcoholic fatty liver disease
ALD: alcoholic liver disease
NOX: NADPH oxidase
NAPQI: N-acetyl-p-benzoquinone imine
GST: glutathione S-transferase
GSSG: oxidized glutathione
COL1A1: collagen1A1
